# N-Glycosylation at Asn291 Stabilizes TIM-4 and Promotes the Metastasis of NSCLC

**DOI:** 10.3389/fonc.2022.730530

**Published:** 2022-03-31

**Authors:** Siyuan Chen, Yuzhen Wang, Wen Liu, Yan Liang, Yingchun Wang, Zhuanchang Wu, Liyun Xu, Xiaohong Liang, Chunhong Ma, Lifen Gao

**Affiliations:** ^1^Department of Immunology, Key Laboratory for Experimental Teratology of Ministry of Education, Shandong Provincial Key Laboratory of Infection and Immunology, School of Basic Medical Sciences, Cheeloo College of Medicine, Shandong University, Jinan, China; ^2^Cell and Molecular Biology Laboratory, Zhoushan Hospital, Zhoushan, China

**Keywords:** NSCLC, TIM-4, N-glycosylation, metastasis, stability

## Abstract

T-cell immunoglobulin domain and mucin domain 4 (TIM-4) is a transmembrane protein that promotes epithelial-mesenchymal transition (EMT), migration and invasion of non-small cell lung cancer (NSCLC) cells. Most transmembrane proteins are modified by N-glycosylation and the importance of protein N-glycosylation in cancer cell metastasis has been well appreciated. However, whether TIM-4 is modified by N-glycosylation and the role of TIM-4 N-glycosylation in NSCLC remains largely unknown. In the current study, we reported that TIM-4 was extensively N-glycosylated at Asn291. After the removal of N-glycosylation, the stability of TIM-4 protein was decreased and TIM-4 was more susceptible to degradation by ER-localized ubiquitin ligase-mediated ERAD. Thus, the expression of TIM-4 on the cell surface was decreased, which suppressed TIM-4-mediated metastasis in NSCLC. In summary, the present study identifies TIM-4 N-glycosylation and its role in NSCLS migration, which would provide a valuable biomarker for developing drugs targeting N-glycosylation at Asn291 on TIM-4.

## Introduction

Lung cancer is the leading cause of cancer deaths worldwide (1). According to WHO classification, lung cancer can be divided into two subtypes: small cell lung cancer and non-small cell lung cancer. Non-small cell lung cancer (NSCLC), accounting for approximately 85% of lung cancer, is the most common malignant tumor (2). Chemotherapy, radiation, and surgical resection are usually combined to intervene NSCLC patients (3). However, the prognosis of these patients remains unsatisfactory so far. Targeted therapy and immunotherapy are considered to alter the landscape of NSCLC treatment. Immune checkpoint inhibitors show good anti-tumor activity, especially in programmed death ligand-1(PD-L1), programmed death receptor-1(PD-1) (4). However, due to individual differences, only a minority of NSCLC patients have improved significantly after receiving immunotherapy. Unfortunately, little progress has been made in the treatment of NSCLC over the past few decades (5, 6). Thus, the discovery of novel treatment strategies is important and urgent for effectively improving prognosis of patients with NSCLC.

Recently, it has been found that epigenetic modification plays an important role in the development of lung cancer, including N-glycosylation (7). N-glycosylation is one of the most frequent post-translational modifications, playing an essential role in determining the structure and function of protein (8). N-Glycosylation is accomplished through collaboration between cellular glycosyltransferases and glycosidase, which add or trim glycans on asparagine (N-linked glycan) residues in polypeptides in the endoplasmic reticulum and the Golgi apparatus (9). N-glycosylation is a highly conserved and most transmembrane proteins are modified by N-glycosylation (10). Emerging evidences indicate that aberrant glycosylation occurs in tumors and is significantly correlated with the progression, metastasis and chemoresistance of tumors (11, 12).N-glycosylation directly affects the biological functions of membrane proteins in cancer cells, such as stability and molecular localization, which might be related to tumor invasion and migration (13). N-glycosylation of PD-L1 in cancer cells was identified in 2016. After the removal of N-glycosylation, the stability of PD-L1 protein was decreased and the degradation rate was accelerated (14). Besides, absence of N-glycosylation in PD-L1 leads to its ER accumulation and ER-associated protein degradation (ERAD) (15). It has been reported that the alterations in glycosylation patterns of many tumor-associated transmembrane glycoproteins such as CD147 are involved in the invasion and migration of cancer cells (16, 17). Therefore, exploitation of altered N-glycosylation of membrane proteins in tumor cells provides novel ideas for the design of novel cancer therapy (18).

T-cell immunoglobulin domain and mucin domain-4 (TIM-4), belonging to the TIM gene family, is a type I transmembrane glycoprotein (19). The molecular structure of TIM-4 protein includes an immunoglobulin-like (IgV) domain, a mucin domain, a transmembrane domain and an intracellular domain (19, 20). TIM-4 is highly expressed on the surface of antigen-presenting cells (APCs), especially activated dendritic cells and resident macrophages (21). However, ectopic expression of TIM-4 has also been detected in parapharyngeal liposarcoma, Langerhans cell sarcoma, colorectal cancer, clear cell renal cell carcinoma and so on (22–24). Our lab has reported that the levels of TIM-4 expression in lung cancer tissues is significantly higher than adjacent tissues. Enhanced TIM-4 expression in NSCLC tissues is negatively related with the prognosis of lung cancer patients (25). Besides, up-regulation of TIM-4 involves in IL-6 promoted metastasis of NSCLC (26). However, the exact site and role of N-glycosylation of TIM-4 in tumors need to be determined. Here, we identified N-glycosylation of human TIM-4 for the first time and investigated its role in NSCLC metastasis.

## Materials and Methods

### Cell Culture

Human NSCLC cell lines A549 and NCI-H1299, human bronchial epithelial (HBE) and human embryonic kidney (HEK293) cells were purchased from the Shanghai Cell Collection of the Chinese Academy of Sciences (Shanghai, China). These cells were grown in DMEM (GIBCO, New York, NY, USA) supplemented with 10% fetal bovine serum (FBS, Gibco-BRL, Grand Island, NY, USA), 100Uml^-1^penicillin and 100 μgml^-1^ streptomycin. Luciferase-A549 cells were infected by concentrated lentivirus carrying TIM-4-Flag gene. Stable cell lines with TIM-4-overexpression or control cells were obtained by screening with a high concentration of puromycin selection (5 μg/mL) for 2 weeks, and subsequently, stable clones were maintained in culture with a low concentration of puromycin (0.5 μg/mL) for tumor bearing in BALB/c nude mice. All the cells were incubated at 5% CO2 and 37°C.

### Plasmids and Transfection

The plasmid pcDNA3-hTIM-4(WT)-HA have been described previously (25). pcDNA3-hTIM-4(N101Q)-HA, pcDNA3-hTIM-4(N291Q)-HA and pcDNA3-hTIM-4(N101/291Q)-HA were constructed by using a KOD-Plus-Mutagenesis Kit (TOYOBO) according to the manufacturer’s instructions. MCherry-Sec61b-C1 was a gift from Jennifer Lippincott-Schwartz (Addgene plasmid #90994; http://n2t.net/addgene:90994; RRID : Addgene 90994) (27).

Cell transfections were performed using Lipofectamine 2000 (Invitrogen) following the manufacturer’s instructions.

### Antibodies

The following antibodies were used: Rabbit anti-TIM-4 (HPA015625, 1:1000 for WB, Sigma-Aldrich), Rabbit anti-E-cadherin (proteintech, 20874-1-AP, 1:1000), Rabbit anti-N-cadherin (proteintech, 22018-1-AP, 1:1000), Rabbit anti-vimentin (proteintech, 10366-1-AP, 1:1000), Mouse anti-HA tag (M180-3, 1:5000 for WB, MBL), Mouse anti-GAPDH (60004-1-Ig, 1:5000 for WB, ProteinTech).

### Invasion and Migration Assays

For migration assays, 6×10^4^ cells were plated in chambers with the non-coated membrane (24-well insert; pore size, 8μm; Corning). For invasion assays, 8×10^4^ cells were plated in chambers with Matrigel-coated membrane (24-well insert; pore size, 8μm; Corning). After a 24h incubation, cells on the upper surfaces of the membrane were removed by a cotton swab and cells on the lower surfaces of the membrane were stained with the crystal violet and counted using an inverted phase-contrast microscope.

### Wound Healing Assay

For the wound-healing assay, the cells were plated in 12-well plates. The cells were scratched using a pipette tip and rinsed to remove debris when the cells were cultured to approximately 100% confluence. Then, the cells were incubated with fresh culture medium containing 1% FBS for 24h. Cell migration were examined at 0 and 24h using an inverted phase-contrast microscope.

### Quantitative Real-Time PCR

Total RNA was extracted from cells, then cDNA synthesis was performed using Revert Aid First Strand cDNA Synthesis Kit (Thermo Fisher Scientific) according to the manufacturer’s instructions for PCR (TIANGEN) or real-time quantitative PCR (TIANGEN) test. The sequences of primers were listed as follows: human (h) E-cadherin-forward (F): 5′-ACAGCCCCGCCTTATGATT-3′,

h-E-cadherin-reverse(R):5′-TCGGAACCGCTTCCTTCA-3′;
h-N-cadherin-F:5′-CAGACATGGAAGGCAATCCCACA-3′,
h-N-cadherin-R:5′-CTGGATGGCGAACCGTCCAGTAGGA-3′;
h-vimentin-F:5′-GCTGAATGACCGCTTCGCCAACT-3′,
h-vimentin-R:5′-GCTCCCGCATCTCCTCCTCGTA-3′;
h-Actin-F:5′-AGTTGCGTTACACCCTTTC-3′,
h-Actin-R:5′-CCTTCACCGTTCCAGTTT-3′;
h-TIM-4-F:5′-ACAGGACAGATGGATGGAATACCC-3′,
h-TIM-4-R:5′-AGCCTTGTG TTTCTGCG- 3′.

### Western Blotting

For immunoblotting, cell lysates were separated by 10% SDS-PAGE and transferred onto PVDF membranes. After the membranes were blocking with 5% Bovine Serum Albumin (BSA, solarbio) for 2h at room temperature, they were incubated with the indicated antibody at 4°C overnight.

### Xenograft Mouse Model of Metastatic Lung Cancer

Migratory ability was determined with lung cancer metastasis xenograft model in male BALB/c (nu/nu) mice (20 ± 2 g, 6–8 weeks old). The study was approved by the institutional guidelines of the Animal Care and Use Committee of Shandong University. 2.0 × 10^6^ A549-LV-TIM-4-Flag or control cells were suspended in 100 μl PBS and inoculated into tail veins of nude mice. 48 hours later, mice were randomly assigned to four groups: (a) LV-NC group with corn oil administration intraperitoneally (n = 5), (b) LV-NC group with tunicamycin (TM) administration intraperitoneally (n = 6), (c) LV-TIM-4 group with corn oil administration intraperitoneally (n = 5) and (d) LV-TIM-4 group with TM administration intraperitoneally (n = 5). The mice were administrated TM (0.1 mg/kg) (28–30) in 100 μl corn oil by intraperitoneal injection twice a week. After 2 weeks, mice were monitored using the IVIS Spectrum *In Vivo* Imaging System (PerkinElmer) of Advanced Medical Research Institute, Shandong University. Mice were intraperitoneally injected with 200 μl of D-luciferin (150 mg/kg body weight, PerkinElmer) and anesthetized with 200 μl 0.5% pentobarbital sodium after 5 minutes. The chest and abdomen of mice were completely exposed, then image calibration and visualization were performed using Living Image 4.2 software (PerkinElmer).

### Statistical Analysis

Statistical analysis was performed using GraphPad Prism 5 Software (San Diego, USA). All data were analyzed by student’s t test (two-tailed). Statistical significances were set at *P < 0.05, **P < 0.01, ***P < 0.001, ****P < 0.0001 and ns represented not significance.

## Results

### TIM-4 Is N-Glycosylated

**Figure 1:**
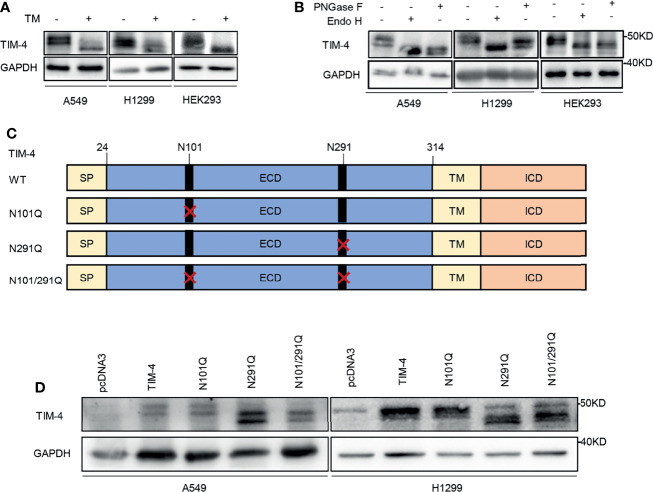
TIM-4 is N-glycosylated. **(A)** Western Blot was used to detect the expression of TIM-4 in lung cancer cell lines and HEK293 cells before and after TM treatment. **(B)** Western Blot was used to detect the expression of TIM-4 in lung cancer cell lines and HEK293 cells before and after PNGase F or Endo H treatment. **(C)** Design of mutated plasmid at the N-glycosylation sites of TIM-4. **(D)** Western blot analysis of the protein expression pattern of WT TIM-4 and N291Q mutants.

TIM-4 recombinant was transfected into lung cancer cell lines and HEK293 cells respectively, and the cells were treated with the N-glycosylation inhibitor TM. Then the cell lysates were used for western blot analysis. The proteins were also treated with PNGase F (recombinant glycosidase) or Endo H (endoglycosidase H), then western blot was performed to detect TIM-4 protein. We found a mobility shift of TIM-4 after TM treatment (**Figure 1A**). Besides, we also found that TIM-4 shifted downward after PNGase F and Endo H treatment compared with their actual sizes (**Figure 1B**). Next, human NSCLC tissue proteins were treated with PNGase F, and A549 cells were treated with the TM the molecular weight of endogenous TIM-4 also had a significant shifted downward (**Figures S1A, B**). These results indicated that TIM-4 was extensively N-glycosylated.

In order to pinpoint the glycosylation sites, we searched for evolutionarily conserved NXT motifs in the TIM-4 amino-acid sequences from different species. Then we used NetGlyc 1.0 to predict N-glycosylation sites of TIM-4 and found two Asparagine (N) residues N101 and N291 on the ectodomain of TIM-4 (**Figure 1C**). To identify the presence of N-glycosylation on these potential residues, each of them was mutated from asparagine (N) to glutamine (Q) separately or together, which were labeled as N101Q, N291Q or N101/291Q. The sequencing results indicated that the N-glycosylation mutants of TIM-4 were constructed successfully for subsequent experiments. Then TIM-4, N101Q, N291Q or N101/291Q were transfected into lung cancer cell lines respectively, and the cell lysates were used for western blot analysis. The results showed that N291Q mutant led to a certain degree of reduction in glycosylation compared with wild type TIM-4. No detectable difference was observed for N101Q mutant. Besides, the N291Q mutant had a similar migration pattern as N101/291Q mutant. Together, these results demonstrated that TIM-4 was exclusively N-glycosylated at Asn291(**Figure 1D**).

### N-Glycosylated TIM-4 Promotes EMT of NSCLC Cells

**Figure 2:**
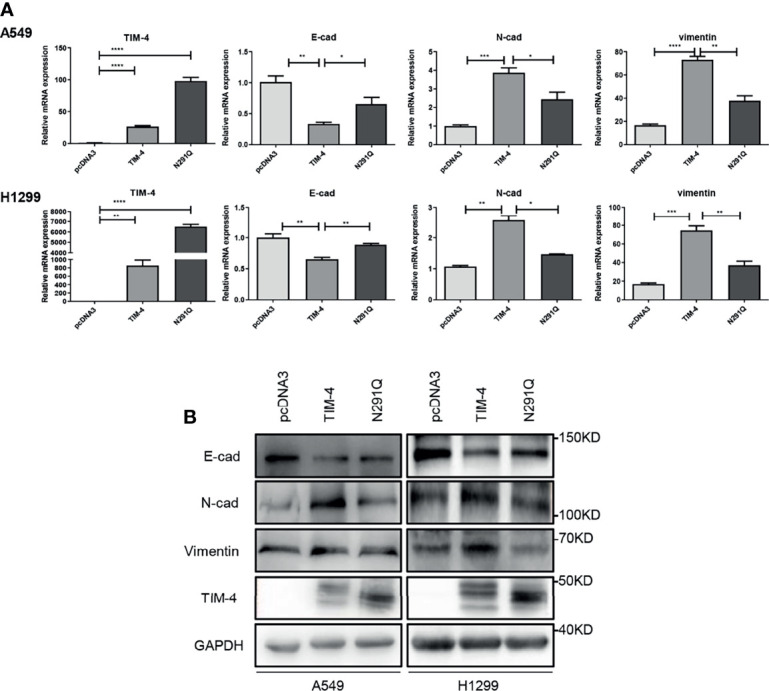
N-glycosylated TIM-4 promotes EMT of NSCLC cells. **(A)**qPCR was used to detect the expression of TIM-4 and EMT related molecules in TIM-4-transfected and N291Q-transfected NSCLC cells. **(B)** Western Blot was used to detect the expression of TIM-4 and EMT related protein in TIM-4-transfected and N291Q-transfected NSCLC cells. All error bars were shown as mean ± SD of three independent experiments. *p < 0.05, **p < 0.01, ***p < 0.001, ****p < 0.0001, by Student’s t-test.

To better understand the significance of N-glycosylation in regulating TIM-4, we further assessed the effect of N-glycosylation on TIM-4 promoted EMT of NSCLC cells. The NSCLC cells were transfected with pcDNA3, TIM-4 or N291Q for 48 h respectively, and the EMT related genes were detected by qPCR and western blot. The results showed that TIM-4 reduced the expression of E-cadherin and increased the expression of N-cadherin and vimentin, while N291Q partially suppress TIM-4-mediated EMT of NSCLC cells (**Figures 2A, B**). The results demonstrated that N-glycosylated TIM-4 played a crucial role in promoting EMT process in NSCLC cells.

### N-Glycosylated TIM-4 Promotes Metastasis of NSCLC Cells

**Figure 3:**
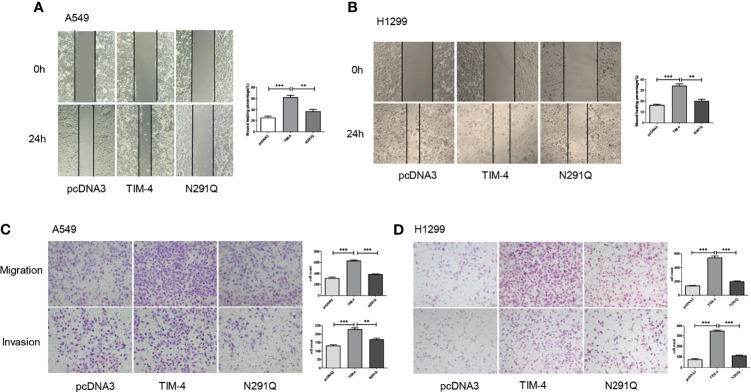
N-glycosylated TIM-4 promotes metastasis of NSCLC cells. Wound healing assay was performed to evaluate the migration abilities of TIM-4-transfected and N291Q-transfected A549 cells **(A)** and H1299 cells **(B)**. Transwell assay was performed to evaluate the migration and invasive abilities of TIM-4-transfected and N291Q-transfected A549 cells **(C)** and H1299 cells **(D)**. (Scale bar:100μm) All error bars were shown as mean ± SD of three independent experiments. **p < 0.01, ***p < 0.001, by Student’s t-test.

Additionally, tumor cells tend to appear much stronger ability in migration and invasion, so the effects of N-glycosylated TIM-4 on the migration and invasion capabilities of NSCLC cells were detected by transwell and wound healing assays. The results showed that the removal of N-glycans at Asn291 markedly reduced NSCLC cells migration and invasion (**Figures 3A–D**). The results showed that N-glycosylated TIM-4 played a crucial role in promoting migration and invasion of NSCLC cells.

### N-Glycosylated TIM-4 Promotes Metastasis of NSCLC Cells *In Vivo*

**Figure 4:**
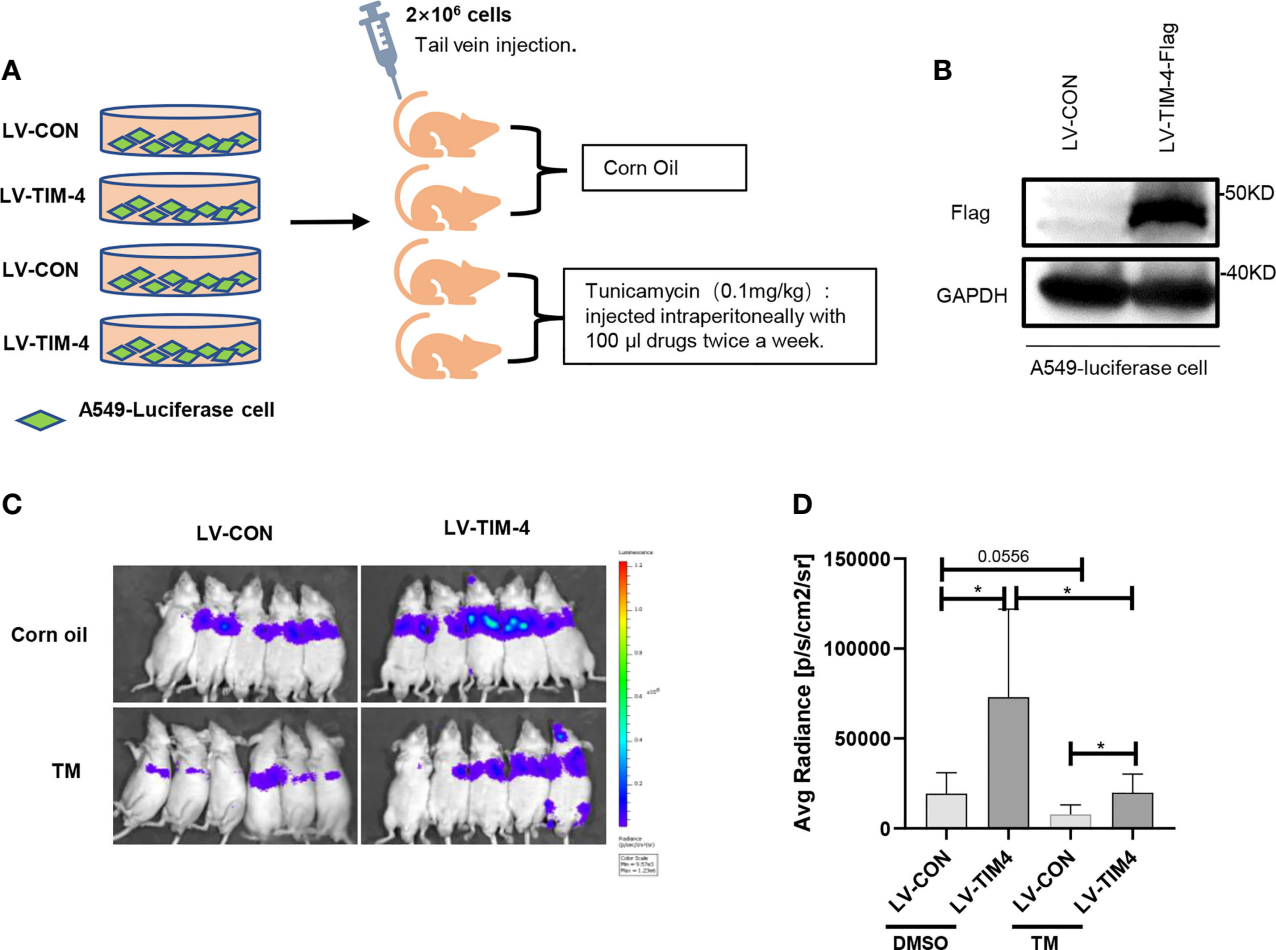
N-glycosylated TIM-4 promotes metastasis of NSCLC cells *in vivo*. **(A)** Experimental flow chart and design. **(B)** Western Blot was used to detect the expression of TIM-4 protein. **(C)** Photographs of nude mice for *in vivo* imaging. **(D)** Statistics of nude mice for *in vivo* imaging. *p < 0.05, by Student’s t-test.

To confirm the findings from the *in vitro* experiments, we evaluated the role of N-glycosylated TIM-4 in NSCLC metastasis *in vivo*. For lung metastasis assay, nude mice were transplanted by 2×10^6^ A549-LV-TIM-4-Flag cells or control cells *via* the tail vein and injected intraperitoneally with 100μl corn oil or TM twice a week, respectively. The experimental flow chart and grouping were shown in **Figure 4A**. Before tumor bearing, TIM-4 expression in A549-LV-TIM-4-Flag cells or control cells was verified by western blot (**Figure 4B**). 2 weeks later, we performed living imaging of mice *in vivo*. Compared with LV-CON group, A549 cells overexpressing TIM-4 carried the powerful metastatic ability and the intensity of fluorescence increased significantly. After treatment with 0.1 mg/kg tunicamycin, the fluorescence was decreased significantly and not well transferred (**Figures 4C, D**). The results showed that N-glycosylated TIM-4 promoted metastasis of NSCLC cells *in vivo*.

### The Stability of TIM-4 Is Decreased After the Removal of N-Glycosylation

**Figure 5:**
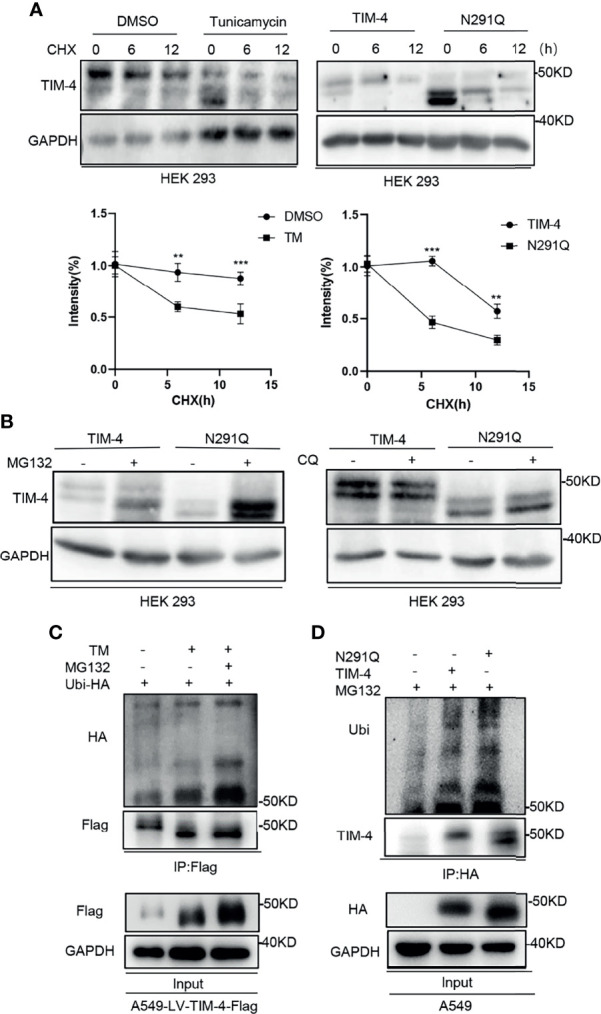
N-glycosylation increases stability of TIM-4. **(A)** Western Blot was used to detect the stability of TIM-4 before and after the removal of N-glycosylation. **(B)** Western Blot was used to detect the degradation pathway of TIM-4 before and after the removal of N-glycosylation. **(C)** Ubi-HA was transfected into A549-LV-TIM-4-Flag cells, and TM or MG132 was used to treat cells. TIM-4 was precipitated by anti-Flag, and Western Blot was used to detect the ubiquitination of TIM-4 before and after the removal of N-glycosylation. **(D)** A549 cells were transfected with pcDNA3, TIM-4-HA or N291Q-HA respectively and treated with MG132. TIM-4 was precipitated by anti-HA, and Western Blot was performed to detect the ubiquitination of TIM-4. **p < 0.01, ***p < 0.001, by Student’s t-test.

It has been reported that N-glycosylation plays a role in maintaining the stability of glycoprotein (31). To identify the role of N-glycosylation in regulating the stability of TIM-4, HEK293 cells were transfected with TIM-4 plasmid and treated with protein synthesis inhibitor cycloheximide (CHX). The CHX-treated cells were further treated with TM or control respectively. The western blot results showed that the turnover rate of TIM-4 protein in the TM group was faster than that of the control group (**Figure 5A**).

Besides, TIM-4 or N291Q were transfected into HEK293 cells and the cells were treated with protein synthesis inhibitor CHX. We found that the degradation rate of TIM-4 protein in N291Q transfected cells was faster than that of wild type TIM-4 (**Figure 5A**). Consistently, TIM-4 were transfected into HEK293 cells and the cells were treated with protein synthesis inhibitor CHX. Quantification of TIM-4 half-life was showed that the half-life of Non-glycosylated TIM-4 protein was shorter than Glycosylated TIM-4 protein (**Figures S1C, D**). The results revealed that the stability of TIM-4 protein was decreased and the degradation rate was accelerated after the removal of N-glycosylation. We further investigated the degradation pathway of TIM-4 after the removal of N-glycosylation. TIM-4 or N291Q were transfected into NSCLC cells and the cells were treated with the proteasome inhibitor MG132 or chloroquine which suppressed the lysosome system pathway. The results showed that TIM-4 protein with N291Q mutation exhibited more accumulation in the presence of MG132, while chloroquine treatment did not alter the expression level of mutated TIM-4 compared with wild TIM-4 (**Figure 5B**). These data suggested that TIM-4 might be degraded in a primarily proteasome-dependent manner after the removal of N-glycosylation.

To test the involvement of 26S proteasome machinery, ubiquitin vector was transfected into A549-LV-TIM-4-Flag cells and we subsequently treated cells with TM and/or proteasome inhibitor MG132. Then the cell lysates were precipitated by Flag antibody and used for western blot analysis. The results showed that non-glycosylated TIM-4 exhibited more ubiquitination in the presence of MG132 (**Figure 5C**). Next, ubiquitin vector was co-transfected with pcDNA3, TIM-4 or N291Q into A549 cells and the cells were treated with MG132. Then the cell lysates were precipitated by HA antibody and used for western blot analysis. The results showed that N291Q mutated TIM-4 exhibited more ubiquitination compared with wild type TIM-4 (**Figure 5D**). These data suggested that TIM-4 might be degraded *via* ubiquitination-dependent proteasome pathway after the removal of N-glycosylation.

### TIM-4 Is Degraded *via* ERAD After the Removal of N-Glycosylation

**Figure 6:**
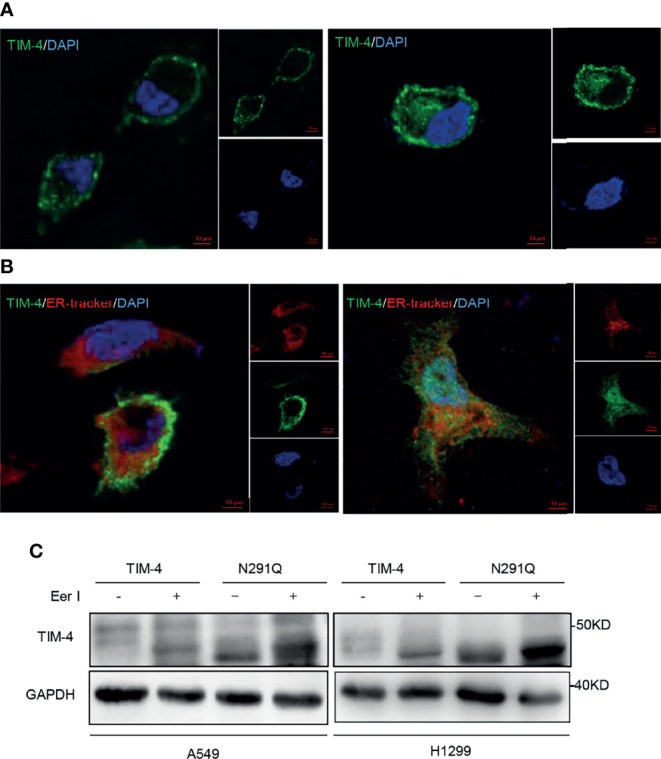
Effects of N-glycosylation on location and degradation pathway of TIM-4. **(A)** Confocal immunofluorescence microscopy was used to detect location of TIM-4 in TIM-4-transfected and N291Q-transfected A549 cells. **(B)** Confocal immunofluorescence microscopy was used to detect colocalization between TIM-4 and ER in TIM-4-transfected and N291Q-transfected A549 cells. (Scale bar:10μm). **(C)** Western Blot was used to detect the degradation pathway of TIM-4.

It has been reported that N-glycosylation plays an important role in the localization of glycoprotein (32). To verify the potential role of N-glycosylation in regulating the localization of TIM-4, TIM-4 or N291Q were transfected into NSCLC cells and cells were observed by immunofluorescence staining assays. The results showed that TIM-4 was primarily localized to the plasma membrane, while N291Q mutated TIM-4 were retained in the cytoplasm (**Figure 6A**). It has been reported that some glycoprotein would be folded incorrectly and displayed a colocalization with the ER (33). To test the localization of TIM-4 after the removal of N-glycosylation, pmCherry-Sec61b-C1 (ER-marker) were co-transfected with TIM-4 or N291Q into NSCLC cells. The immunofluorescence staining assay showed that the localization of N291Q mutated TIM-4 was changed and co-localized with the ER-tracker compared with wild type TIM-4 (**Figure 6B**).

It has been reported that some glycoproteins are folded incorrectly and sequentially degraded by ERAD pathway (ER-associated degradation). In order to test the degradation pathway of TIM-4 after the removal of N-glycosylation, TIM-4 or N291Q were transfected into NSCLC cells and the cells were treated with Eer I, inhibitor of ERAD, then the cell lysates were used for western blot analysis. The results showed that N291Q mutated TIM-4 exhibited more accumulation in the presence of Eer I compared with wild type TIM-4. These results suggested that TIM-4 might be degraded by ERAD pathway after the removal of N-glycosylation (**Figure 6C**).

## Discussion

TIM-4, a type I transmembrane protein, is expressed on many types of cells, including tumor-associated myeloid cells and cancer cells. TIM-4 on tumor-associated myeloid cells directly interacts with adenosine monophosphate activating kinase-α1(AMPKα1) to activate autophagy-mediated degradation of ingested tumors, resulting in reduced antigen presentation and impaired cytotoxic T lymphocyte (CTL) response. Thus, targeting of the TIM-4-AMPKa1 interaction would be effective to augment antitumor immunity and improve cancer chemotherapy (20). Recently, it has been reported that TIM-4 is specifically expressed in subcutaneous panniculitis T-cell lymphoma (SPTCL) malignant cells, indicating that TIM-4 may be a potential novel marker for SPTCL (34).Indeed, TIM-4 has been reported as a potential target for cancer diagnosis and therapy (35).Besides, TIM-4 is detected in several kinds of cancer including glioma, colorectal cancer, parapharyngeal liposarcoma, and so on. Consistently, our studies show that the expression of TIM-4 in NSCLC is significantly higher than adjacent tissues and increased expression of TIM-4 promotes metastasis of NSCLC. However, the mechanism of TIM-4-induced NSCLC metastasis remains unclear.

N-glycosylation is a highly conserved and the most common post-translational modification, especially for transmembrane proteins (36). It has not been reported whether TIM-4 is modified by N-glycosylation in NSCLC cells. Glycosylation of proteins often leads to heterogeneous patterns on western blots. Intriguingly, varied forms of protein band of TIM-4 were observed in human NSCLC tissues (**Figure S1E**).

We used NetGlyc 1.0 to predict N-glycosylation sites of TIM-4 and Asn101 and Asn291 which might be potential N-glycosylation sites of TIM-4. Additionally, predicted N-glycosylation sites in human are also evolutionarily conserved (**Figure S1F**). With this, the N-glycosylation of TIM-4 raised our concern. Next, we demonstrated that the existence of N-glycosylation of TIM-4 in NSCLC cells. And our site-directed mutagenesis studies confirmed that TIM-4 was extensively N-glycosylated at Asn291. We mentioned that the levels of messenger mRNA of the mutant were significantly higher than those of the wild type variant. We are not sure whether mutation of TIM-4 at Asn291 would affect the stability of TIM-4 mRNA or regulation of miRNA, which requires further investigation in the future.

Because N-glycosylation is critical for the function of protein, we sought to explore the glycosyltransferases involved in the N-glycosylation of TIM-4. The Cancer Genome Atlas (TCGA) dataset was used to analyze and predict glycosyltransferases that are highly correlated with TIM-4 expression, among which the expression of FUT4 was correlated positively with TIM-4 (**Figure S2A**). QPCR analysis further showed that FUT4 was correlated with TIM-4 in human NSCLC tissues (**Figure S2B**), indicating that FUT4 may mediate the N-glycosylation of TIM-4. However, more evidences are required to support the idea.

Accumulating evidences have indicated that aberrant N-glycosylation occurs frequently in tumors and is closely associated with cancer metastasis (37–39). Our previous studies indicated that TIM-4 overexpression significantly promoted EMT process of NSCLC cells (26). In this study, we also attempted to assay the role of TIM-4 N-glycosylation in TIM-4-mediated EMT process of NSCLC cells. Based upon our current findings, TIM-4 N-glycosylation promotes EMT process of NSCLC cells. It has been reported that EMT plays an important role in invasion and migration of cancer cells (40, 41). We further confirmed that N-glycosylated TIM-4 promoted metastasis of NSCLC cells *in vitro* and *in vivo*. However, the related mechanism of TIM-4 N-glycosylation modulating EMT and NSCLC metastasis requires to be further investigated.

It has been reported that N-glycosylation affects the function of protein by affecting the stability of glycoprotein (42). In order to explore the effect of N-glycosylation at Asn291 on TIM-4 stability, we detected the degradation ability of TIM-4. Then, we found that the stability of TIM-4 protein was decreased and the degradation rate was accelerated after the removal of N-glycosylation. Besides, we also confirmed that TIM-4 was degraded *via* ubiquitination-dependent proteasome pathway after the removal of N-glycosylation. Increasing evidences indicate that N-glycosylation is essential for glycoprotein localization (33, 43). In this study, we compared the localization of glycosylated and non-glycosylated TIM-4. we found that glycosylated TIM-4 was mostly distributed to the cell membrane, while non-glycosylated TIM-4 was trapped in the cytoplasm. Besides, non-glycosylated TIM-4 was largely retained in the ER and co-localized with the ER-tracker compared with glycosylated TIM-4. It has been reported that N-glycosylation plays an important role in ERAD of proteins by cytosolic proteasomes (44). When the protein was misfolded, or when abnormal glycan structure is present, the proteins would be degraded by ERAD pathway (45, 46). As expected, we found that TIM-4 was degraded by ERAD pathway after the removal of N-glycosylation.

**Figure 7:**
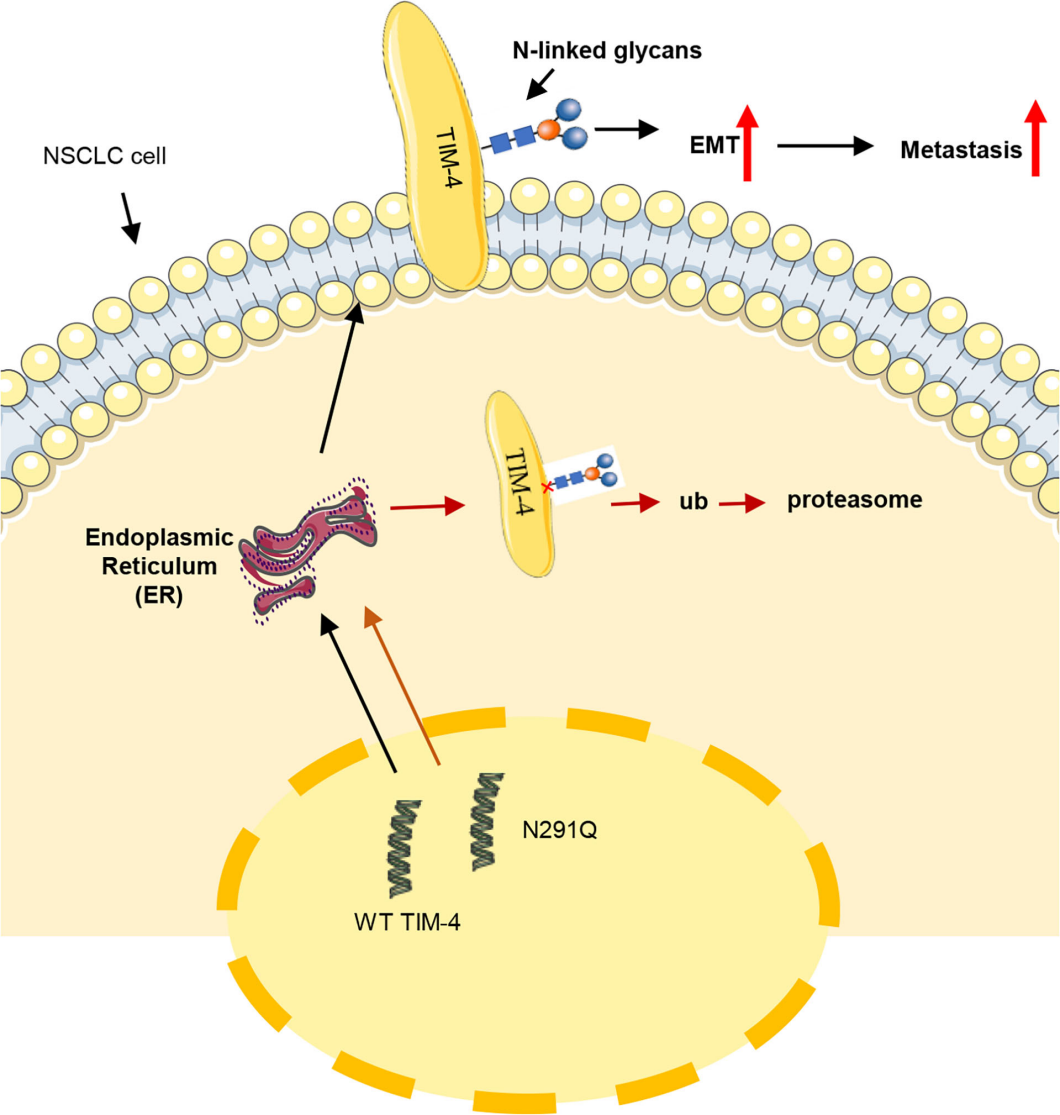
Schematic representation of the role of TIM-4 N-glycosylation in NSCLC metastasis. TIM-4 is N-glycosylated at Asn291 and TIM-4 promotes NSCLC metastasis by modifying N-glycans at Asn291. When the N-glycosylation of TIM-4 is removed, the stability of TIM-4 protein is decreased and TIM-4 is more susceptible to degradation by ER-localized ubiquitin ligase-mediated ERAD. Thus, the function of TIM-4-mediated metastasis of NSCLC cells was suppressed.

Based on the findings of this study, we propose a model to explain the role of N-glycosylation in TIM-4 mediated NSCLC metastasis and the related mechanism (**Figure 7**). TIM-4 was identified to be extensively N-glycosylated at Asn291 and N-glycosylated TIM-4 promotes metastasis of NSCLC cells. After the removal of N-glycosylation, the stability of TIM-4 protein was decreased and TIM-4 was more susceptible to degradation by ER-localized ubiquitin ligase-mediated ERAD. Thus, the expression of TIM-4 on the cell surface was decreased and the function of TIM-4-mediated metastasis of NSCLC cells was suppressed.

In summary, our findings potentially shed light on the mechanism of N-glycosylated TIM-4 and provide a valuable biomarker for developing drugs targeting N-glycosylation at Asn291 on TIM-4, which may offer a potential new strategy for treating NSCLC in clinical.

## Data Availability Statement

The original contributions presented in the study are included in the article/**Supplementary Material**. Further inquiries can be directed to the corresponding author.

## Ethics Statement

The studies involving human participants were reviewed and approved by the Ethics Committee of Shandong University School of Basic Medical Sciences. The patients/participants provided their written informed consent to participate in this study. The animal study was reviewed and approved by the Animal Care and Use Committee of Shandong University School of Basic Medical Sciences.

## Author Contributions

LG supervised all the subjects and gave an elaborate guidance for this project. LG and SC designed the experiments and wrote the manuscript. SC, YZW, WL, YL, YCW, ZW, and LX performed the experiments and analyzed the data. CM and XL helped to design the experiments. All authors contributed to the article and approved the submitted version.

## Funding

This work was supported by the National Natural Science Foundation of China (81971480, 81670520), Joint fund project of Natural Science Foundation of Shandong Province (ZR2019LZL013), Major Basic Research Project of Shandong Natural Science Foundation (ZR2020ZD12), National Key Research and Development Program (2018YFE0126500), Shandong Provincial Key Innovation project (2018YFJH0503) and Shandong University multidisciplinary research and innovation team of young scholars (2020QNQT001).

## Conflict of Interest

The authors declare that the research was conducted in the absence of any commercial or financial relationships that could be construed as a potential conflict of interest.

## Publisher’s Note

All claims expressed in this article are solely those of the authors and do not necessarily represent those of their affiliated organizations, or those of the publisher, the editors and the reviewers. Any product that may be evaluated in this article, or claim that may be made by its manufacturer, is not guaranteed or endorsed by the publisher.

## Supplementary Material

Supplementary Figure 1 TIM-4 is heterogeneous and evolutionarily conserved in NSCLC. **(A)** Western Blot was used to detect the expression of TIM-4 in lung cancer tissues before and after PNGase F treatment. **(B)** Western Blot was used to detect the expression of endogenous TIM-4 in lung cancer cell line before and after TM treatment. **(C)** Western Blot was used to detect the half-life of TIM-4.Quantification of TIM-4 half-life was shown in **(D)**. **(E)** Western Blot was used to detect the expression of TIM-4 in lung cancer tissues. **(F)** Sequence alignment of the TIM-4 amino acid sequences from different species.

Supplementary Figure 2 FUT4 was correlated with TIM-4 in human NSCLC tissues. **(A)** Correlation between the RNA expression of TIM-4 and FUT4 in TNBC. **(B)** Correlation between the protein expression of TIM-4 and the RNA expression of FUT4 in lung cancer patient tissues.
