# Supplementary figures and images for: N-Glycosylation at Asn291 Stabilizes TIM-4 and Promotes the Metastasis of NSCLC
Source: Front Oncol. 2022 Mar 31;12:730530. doi: 10.3389/fonc.2022.730530 (PMC9008408; doi:10.3389/fonc.2022.730530)

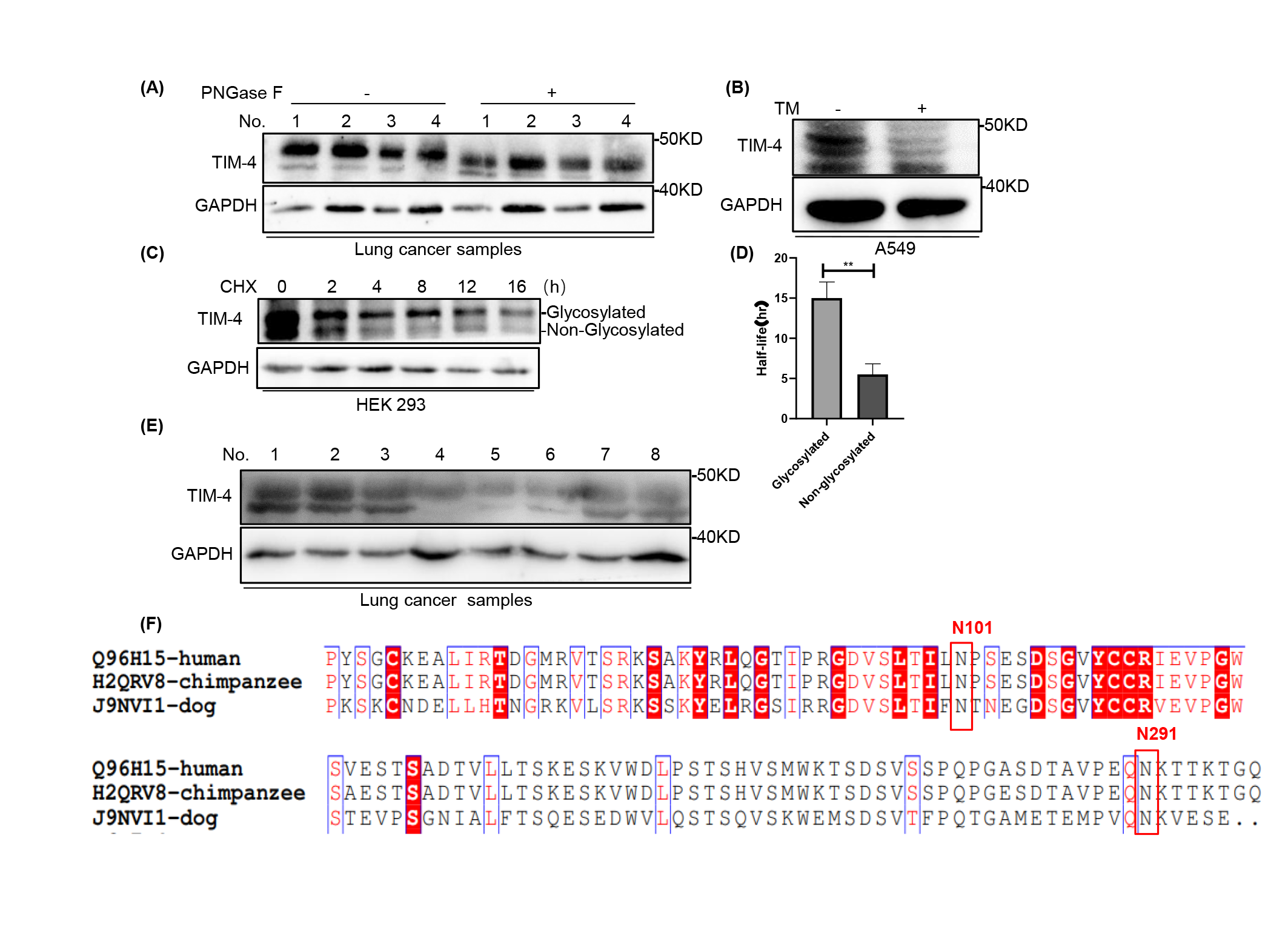

Supplement: Supplementary Figure 1 — TIM-4 is heterogeneous and evolutionarily conserved in NSCLC. (A) Western Blot was used to detect the expression of TIM-4 in lung cancer tissues before and after PNGase F treatment. (B) Western Blot was used to detect the expression of endogenous TIM-4 in lung cancer cell line before and after TM treatment. (C) Western Blot was used to detect the half-life of TIM-4.Quantification of TIM-4 half-life was shown in (D). (E) Western Blot was used to detect the expression of TIM-4 in lung cancer tissues. (F) Sequence alignment of the TIM-4 amino acid sequences from different species. [file Image_1.tif]

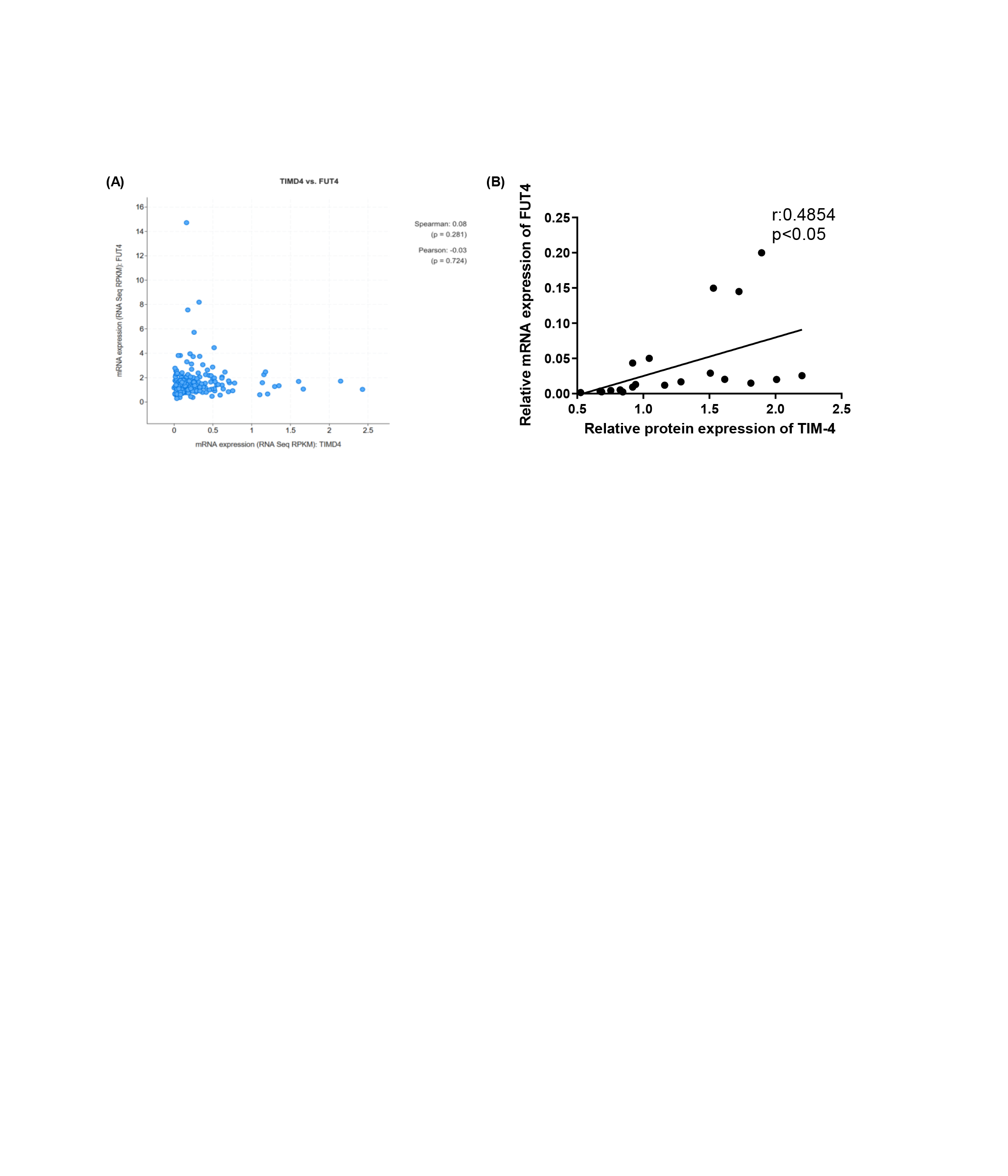

Supplement: Supplementary Figure 2 — FUT4 was correlated with TIM-4 in human NSCLC tissues. (A) Correlation between the RNA expression of TIM-4 and FUT4 in TNBC. (B) Correlation between the protein expression of TIM-4 and the RNA expression of FUT4 in lung cancer patient tissues. [file Image_2.tif]
